# Prioritisation criteria for the selection of new diagnostic technologies for evaluation

**DOI:** 10.1186/1472-6963-10-109

**Published:** 2010-05-05

**Authors:** Annette Plüddemann, Carl Heneghan, Matthew Thompson, Nia Roberts, Nicholas Summerton, Luan Linden-Phillips, Claire Packer, Christopher P Price

**Affiliations:** 1 Oxford Centre for Monitoring and Diagnosis, Department of Primary Health Care, University of Oxford, Oxford OX3 7LF, UK; 2 National Horizon Scanning Centre, Department of Public Health, Epidemiology and Biostatistics, The University of Birmingham, Edgbaston, Birmingham, B15 2TT, UK

## Abstract

**Background:**

Currently there is no framework for those involved in the identification, evaluation and prioritisation of new diagnostic technologies. Therefore we aimed to develop prioritisation criteria for the assessment of new diagnostic technologies, by gaining international consensus on not only which criteria should be used, but also their relative importance.

**Methods:**

A two-round Delphi process was used to generate consensus amongst an international panel of twenty-six experts on priority criteria for diagnostic health technology assessment. Participants represented a range of health care and related professions, including government, industry, health services and academia.

**Results:**

Based on the responses to the first questionnaire 18 criteria were placed into three categories: high, intermediate and moderate priority. For 16 of the 18 criteria, agreement with the categorisation of the criteria into the high, intermediate and moderate categories was high at ≥ 70% (10 had agreement ≥ 80%). A further questionnaire and panel discussion reduced the criteria to 16 and two categories; seven were classified as high priority and nine intermediate.

**Conclusions:**

This study proposes an objective structure of prioritisation criteria to use when assessing new diagnostic technologies, based on an expert consensus process. The value of these criteria is that no one single component should be used as the decisive driver for prioritisation of new diagnostic technologies for adoption in healthcare settings. Future studies should be directed at establishing the value of these prioritisation criteria across a range of healthcare settings.

## Background

A correct diagnosis is central to guiding choice of treatment, monitoring the effects of that treatment, as well as determining prognosis. Diagnosis involves both clinical skills (e.g. physical examination), as well as techniques to measure physiological parameters (e.g. blood pressure), and biochemical, haematological, other pathological and radiological investigations. New diagnostic technologies are continually being developed and marketed, while technologies that are currently used in hospital or traditional laboratory settings are increasingly being repackaged as point of care devices. However, improvements in diagnostic technologies do not inevitably translate to benefits to patient care [1]. For example, a new test may not have a clearly defined role in an existing diagnostic pathway, or may simply contribute to diagnostic uncertainty. Unlike randomised trials of interventions, which have a control arm, most studies of diagnostic accuracy do not compare the outcome from the use of the new test with existing tests [2].

In order for health care purchasers and providers to assess the importance and role of a new diagnostic technology in the diagnostic pathway, it is vital to have a process that can identify which technologies require more detailed or formal assessment, such as technology assessments or evidence-based summary reports. Simply using the efficacy data of a new diagnostic technology derived from a clinical study to determine adoption in the healthcare setting is inadequate [3]. This approach does not take into account all the factors influencing adoption of a test, such as the clinical setting, disease prevalence and proposed use.

Prioritising health technologies for further assessment, particularly for therapeutic interventions, is well established among international health technology assessment (HTA) bodies. Priority setting has involved both quantitative methods or scoring systems [4], as well as consensus guidelines. Quantitative models have been developed, for example by the Institute of Medicine in the USA, who developed a specific quantitative method to calculate priority scores based on criterion weight, as well as scores proposed by the Committee on Priorities for Assessment and Reassessment of Health Care Technologies [4], and the Technology Assessment Priority-Setting System (TAPSS) developed by the Council on Health Care Technologies [5]. In Europe, the EEC-funded EUR-ASSESS project for the coordination of HTA activities produced a set of guidelines for the priority setting of HTA projects [6]. Indeed, a recent systematic review identified 12 different priority setting frameworks used among 11 international HTA agencies, with a total of 59 different priority setting criteria [7]. Although the existing frameworks are intended to be applied to all technologies, there is a paucity of guidance on methods for prioritising diagnostic technologies in particular.

There are several reasons why diagnostic technologies require prioritisation criteria that are distinct from existing frameworks. Diagnosis usually involves a pathway in which a new technology may have a variety of roles such as replacing an existing test, triaging, or as an add-on test. In addition diagnostic accuracy may vary widely between different clinical settings and populations due to variations in prevalence and spectrum of disease. Key issues that are important in the evaluation of new diagnostic technologies have been proposed previously - these fitted into three broad domains, those related to the disease or target condition, the new diagnostic technology itself, and the impact of the diagnostic technology [8]. To address the important gap in the technology prioritisation of diagnostic technologies we aimed to build on these criteria, by refining them and including additional criteria. We also aimed to assess the criteria systematically by developing an international consensus on not only which criteria should be used to prioritise diagnostic technologies, but also their relative importance.

## Methods

We used the Delphi method, which is an objective process that gathers consensus opinion from a panel of experts through an iterative questionnaire process interspersed with controlled opinion feedback [9]. Panels generally involve 10 to 50 members and experts who are anonymous, in that other panel members do not know their identity at the time of data collection. The Delphi method has been used extensively in developing criteria frameworks [10,11]. No ethical approval was required for this study.

We identified an international group of experts as follows: (1) membership of early awareness and alert networks (e.g. The International Information Network on New and Emerging Health Technologies [EuroScan]), (2) recommendation from researchers in the field of health technology assessment, (3) contacts in diagnostic technology industries, (4) contacts in government bodies tasked with health technology assessment, and (5) recommendations from researchers and providers in the field of primary health care and diagnosis. Experts were contacted by email and invited to contribute to the study using either email or a web-based format and were from a variety of health care sectors and related professional disciplines (Table 1).

**Table 1:** The Panel: Sectors and Main Professional Roles

Panel	Number
***Sector***	

Government	3

Academic	8

Industry	7

Health Services	11

	

***Main Professional Role***	

Health care professional	7

Research	9

Policy	2

Teaching	2

Management	7

Health Technology Assessment	5

Marketing	2

Consultancy	1

	

***Total number of participants***	**26**

The first questionnaire used prioritisation criteria developed by Summerton [8], which were compiled into an initial questionnaire following preliminary discussions amongst a small group of experts. The initial questionnaire consisted of 18 criteria, which were grouped into those pertaining to (1) the disease or target condition, (2) the new diagnostic technology, and (3) the impact of the diagnostic technology (Table 2). Participants were asked to rate the importance of, or their level of agreement with, each criterion using a seven-point Likert scale, where 1 indicated low and 7 high importance or levels of agreement. Open comments or clarifications on each item, as well as general comments and suggestions regarding items overlooked in the questionnaire were solicited. In total 26 respondents participated in the first questionnaire.

**Table 2:** Criteria appraised by experts: Round 1 Questionnaire

	Percent ranking 6 or 7
**Regarding the disease or target condition**	

1. The disease or target condition to which the diagnostic technology will be applied can be clearly defined.	73%

2. The prevalence or incidence of the disease or target condition.	58%

3. The potential that the technology will have an impact on morbidity and/or mortality of the disease or target condition.	88%

4. The relevance of the disease or target condition to current regional or national health policies and/or priorities.	23%

5. The accuracy of the current diagnostic approach for the disease or target condition is problematic.	65%

6. There is variation in treatment or patient outcomes resulting from current diagnostic variability.	58%

7. The current diagnostic pathway for the disease or target condition could be improved by obtaining information in a less risky fashion or in a manner more acceptable to patients.	65%

**Regarding the new diagnostic technology**	

8. The new technology has a clearly defined role in the diagnostic pathway, e.g. replacing an existing test, as a triage tool, or after the diagnostic pathway as an add-on test.	58%

9. The new technology improves the ability to rule out the disease or target condition.	77%

10. The safety profile of the new technology has been established.	62%

11. There is evidence of test accuracy in the setting in which the new diagnostic technology will be applied.	70%

12. The new technology improves the ability to rule in the disease or target condition.	62%

13. The new technology would enhance diagnostic efficiency or be more cost effective than the current diagnostic approach.	65%

14. It would be feasible to change current practice to incorporate this technology (e.g. additional training, infrastructure, or quality control).	46%

15. The new technology reduces the number of people falsely diagnosed with the disease or target condition.	85%

**Regarding the impact of the diagnostic technology**	

16. Improved diagnostic precision using the technology would lead to improvement(s) in the delivery of treatment (e.g. shorter time to initiating treatment, reduction in morbidity or mortality).	81%

17. The new diagnostic technology will decrease workload in managing the disease or target condition.	35%

18. The cost-effectiveness of the new technology compared to existing standard practice.	50%

Responses to the first questionnaire were categorised according to the proportion of respondents that ranked criterion as a 6 or 7 on the Likert scale. Based on this analysis three priority groups were created: (1) high priority, at least 70% of respondents ranked 6 or 7, (2) intermediate priority, 50-69% of respondents, and (3) moderate priority, less than 50% of respondents.

A second questionnaire was then developed, placing the criteria in the aforementioned three priority groups. The experts were informed of the method used to group the criteria and were asked to indicate whether they agreed or disagreed with the placement of each criterion into its respective priority group. If they disagreed with the placement of a criterion, they were asked to suggest which category the criterion should be placed into (high, intermediate or moderate) and provide clarifying comments for their opinion. General comments were also invited. The final questionnaire responses were reviewed at a focus panel meeting of a smaller group of experts.

## Results

The 26 respondents represented a broad section of the health care sector: government (10%), industry (24%), academic (28%) and health services (38%), with diverse professional roles (Table 1). In some instances participants had more than one professional role and/or worked for different sectors (e.g. health care services and academic).

### Round 1 Questionnaire

For 5 of the 18 criteria in the first questionnaire (Table 2; criteria 1, 3, 9, 15, 16), we found a high level of consensus existed as to their prioritisation importance. For example, 88% of respondents assigned a high importance to "*The potential that the technology will have an impact on morbidity and/or mortality of the disease*" and 85% to "*The new technology reduces the number of people falsely diagnosed with the disease or target condition*". Although 73% of respondents ranked "*The disease or target condition to which the diagnostic technology will be applied can be clearly defined" *as a 6 or 7 on the scale of importance, 13%, primarily from the academic sector, considered this criterion unimportant, ranking it as 1 or 2 on the Likert scale.

Seven criteria (Table 2; criteria 2, 4, 6, 8, 14, 17, 18) showed a substantial variation in ranking of their importance. For example, 46% of respondents assigned a low level of importance to "*The relevance of the disease or target condition to current regional or national health policies and/or priorities*", with 23% scoring a 1 or 2, whereas 23% rated this criterion as important, scoring a 6 or 7. Respondents ranking this criterion as being of low importance (1 or 2 on the Likert scale) came from academic, industry and health services sectors, whereas respondents from the government sector gave this criterion more importance (4 to 6 on the Likert scale). Reasons provided for rating this criterion low included: regional and national policies are sometimes out-of-date, priorities can be skewed by political pressure, and it is most important how much morbidity and mortality can be avoided rather than whether it is a government priority. However some respondents from industry and academia ranked this criterion as a 7 in terms of its importance. Regarding "*The prevalence or incidence of the disease or target condition*", respondents commented uncommon conditions could still be important to consider for research. "*It would be feasible to change current practice to incorporate this technology (e.g. additional training, infrastructure, or quality control)" *showed the most rankings of 5, indicating that this criterion was deemed somewhat important, but not the most important.

"*The potential that the technology will have an impact on morbidity and/or mortality of the disease*" scored the highest mean Likert value of 6.5 and the criterion that "*The relevance of the disease or target condition to current regional or national health policies and/or priorities*" had the lowest mean value of 4.2. Based on the responses to the round 1 questionnaire, we categorised the criteria into high (70% or more respondents ranked criteria as 6 or 7 on the scale of importance), intermediate (50-69% ranked criteria as 6 or 7 on the scale of importance) and moderate priorities (less than 50% ranked criteria as 6 or 7 on the scale of importance) for the second round questionnaire.

### Round 2 Questionnaire

A total of 24 (92%) of those who participated in the first questionnaire responded to the second questionnaire. For 16 of the 18 criteria, agreement with the categorisation of the criteria into the high, intermediate and moderate categories was high at ≥ 70% (10 had agreement ≥ 80%). There was low agreement for two criteria: "*The prevalence or incidence of the disease or target condition*" (54% agreement) and "*There is variation in treatment or patient outcomes resulting from current diagnostic variability*" (67% agreement). For the latter, a number of respondents commented it should be moved from the intermediate to high priority groups, since practice variation is an important driver of prioritisation and variation in outcomes is a clear sign that the current diagnostic process is not fit for purpose. For "*The prevalence or incidence of a disease or target condition*", several experts disagreed with its placement into the intermediate category and suggested it should have a high priority. The main reason given for this was technologies that have an impact on highly prevalent diseases would have the greatest impact within the health care system. However, other experts felt this criterion should be moved to moderate priority, as they considered that it would not be a useful criterion in itself if the technology did not meet any of the other criteria.

Several experts also suggested moving the criteria placed into the "moderate priority" category into either the high or intermediate priority categories. Based on the subsequent panel discussion, there did not appear to be a clear reason to differentiate between intermediate and moderate priority categories, so these were combined. Two criteria were removed from the final list as several respondents commented that they overlapped with other criteria. These were the criteria relating to cost-effectiveness and workload, which were already addressed by "*The new technology would enhance diagnostic efficiency or be more cost effective than the current diagnostic approach*". The latter criterion was then moved to high priority, in line with expert suggestions. The final list consisted of 16 criteria, seven of which were considered high priority and nine of intermediate priority (Table 3).

**Table 3:** Proposed Criteria for the Prioritisation of Diagnostic Technologies

	Does the technology meet the criterion?		
***High Priority***	**Yes**	**No**	**Unsure**

1. The potential that the technology will have an impact on **morbidity and/or mortality **of the disease or target condition.			

2. The new technology **reduces the number of people falsely diagnosed **with the disease or target condition.			

3. Improved diagnostic precision using the technology would lead to **improvement(s) in the delivery of treatment **(e.g. shorter time to initiating treatment, reduction in morbidity or mortality).			

4. The new technology improves the ability to **rule out **the disease or target condition.			

5. The disease or target condition to which the diagnostic technology will be applied can be **clearly defined**.			

6. There is evidence of test accuracy in the **setting **in which the new diagnostic technology will be applied.			

7. The new technology would **enhance diagnostic efficiency **or be more cost effective than the current diagnostic approach.			

***Intermediate Priority***			

1. The **prevalence or incidence **of the disease or target condition.			

2. The accuracy of the **current diagnostic approach **for the disease or target condition is problematic.			

3. There is **variation **in treatment or patient outcomes resulting from current diagnostic variability.			

4. The current diagnostic pathway for the disease or target condition could be improved by obtaining information in a **less risky fashion **or in a manner more acceptable to patients.			

5. The **safety profile **of the new technology has been established.			

6. The technology improves the ability to **rule in **the disease or target condition.			

7. The new technology has a **clearly defined role **in the diagnostic pathway, e.g. replacing an existing test, as a triage tool, or after the diagnostic pathway as an add-on test.			

8. The relevance of the disease or target condition to current **regional or national health policies and/or priorities**.			

9. It would be **feasible to change **current practice to incorporate this technology (e.g. additional training, infrastructure, or quality control).			

## Discussion

In this study we have developed prioritisation criteria for the evaluation of new diagnostic technologies. With the aid of a two-round Delphi consensus method, which sought the opinions of 26 experts, 16 criteria were agreed, of which seven were classified as high priority and nine as intermediate priority. To our knowledge this is the first study to address prioritisation criteria for diagnostic technologies using the Delphi method.

This study was designed to canvass opinions from a range of experts from industry, academia, government and health services. The aim of such a process is to obtain a range of opinions from diverse perspectives and, unlike quantitative surveys, does not rely on a large sample to determine outputs with confidence. Although our list of experts is not exhaustive, the group provided a wide range of views and represented several different sectors. The prioritisation criteria presented are therefore not dependent on the views of professionals from one specific constituency or working in a particular health care system or country. However, we acknowledge that the generalisability of the findings of the Delphi consensus approach may be limited by the relatively small number of participants, who may have specific views or agendas. Further studies to verify and refine our results in different or extended groups of participants should be considered.

A wide range of quantitative and qualitative prioritisation criteria for health technologies have been published by committees and organisations involved in health care [5,7]. Generally the criteria lists share three main elements: (1) clinical impact, (2) economic impact and (3) budget impact. A weighted benefit score coupled with cost for prioritising health technologies at the primary care trust level was developed by Wilson et al [12]. The authors applied this score to six proposed services and found that it was practical, however ultimately the primary care trust was not able to use the results of the score as the sole criteria to prioritise which services to fund. Indeed, two of the prioritised services did not receive funding, indicating that the criteria were inadequate. A systematic review of 12 priority-setting frameworks from 11 agencies in 10 countries highlighted differences across HTA agencies regarding categorisation, scoring and weighting of criteria [7]. The review showed that quantitative rating methods and cost benefit considerations for priority setting were seldom used.

Although criteria have been developed for priority setting of new health technologies for early assessment, these are generally applied to novel therapeutic agents and interventions [13]. Selection criteria also differ significantly depending on the early awareness programme, and prioritisation is frequently implicit and undocumented [13,14]. The requirements for diagnostic technologies are somewhat different. For example, while the prevalence or incidence of a disease is a primary criterion listed in many of the existing prioritisation frameworks, the consensus emerging from our study indicates that, in terms of diagnostic technologies, this criterion carries less weight: a test for a relatively uncommon disease (e.g. pancreatic cancer) may still be very important. Diagnostic technologies may also have different outcomes, for example either ruling in or ruling out a disease. Here it emerged that ruling out a disease is of higher priority in diagnosis, although this may not be the case in all clinical settings: in high acuity situations, such as critical care, ruling in a disease may be more important.

Although in some cases there was disagreement amongst the panel regarding the placement of criteria into their respective categories, overall the level of consensus was high. The criteria set out in this study should be relevant to those involved in identification, evaluation and prioritisation of new diagnostic technologies at national, regional or local levels. Our aim is to provide a framework for the selection of new diagnostic technologies for in-depth assessment or implementation, based on how many and the extent to which the listed criteria are satisfied and whether they fall into the high or intermediate priority category. This could be achieved by assessing which criteria are met via a check-list (Table 3). One strategic use of such a checklist, adopted by our Diagnostic Technology Horizon Scanning Centre [15], is to highlight areas where there is lack of evidence and where further research is required. Different specialities may also apply different weights to the criteria, depending on their priorities. These criteria could be adopted by the 'Evaluation Pathway Programme for Medical Technologies' recommended in the NHS Next Stage review and currently being established by UK National Institute for Clinical Excellence, to which some diagnostic technologies will be subject. In addition, they would potentially also be applicable to the Agency for Healthcare Research and Quality's (AHRQ) proposed new Horizon Scanning System in the USA [16]. Future studies should be directed at establishing the value of these prioritisation criteria in such settings. An important challenge will be to identify the supporting evidence base required to assess the high priority criteria in particular.

## Conclusions

The prioritisation criteria presented in this study provide an objective structure for the assessment of new and emerging diagnostic technologies. In effect the value of these criteria is that no one single component should be used as the decisive driver for prioritisation of new diagnostic technologies for adoption in healthcare settings, irrespective of the context. We encourage diagnostic technology programmes to evaluate further the value of these criteria.

## Competing interests

The authors declare that they have no competing interests.

## Authors' contributions

All authors conceived of the study, participated in its design and coordination and helped to draft the manuscript. All authors read and approved the final manuscript.

## Appendix 1: List of expert participants

Anne Mackie, National Screening Committee, UK

Anthony Harnden, Department of Primary Health Care, University of Oxford, UK

Anthony James, NHS Institute for Innovation and Improvement, UK

Birgitte Bonnevie, Danish Centre for Evaluation and Health Technology Assessment, Denmark

Brian Shine, Department of Clinical Biochemistry, University of Oxford, UK

Carl Heneghan, General Practice and Department of Primary Health Care, University of Oxford, UK

Christopher P Price, Department of Primary Health Care, University of Oxford, UK

Danielle Freedman, Royal College of Pathologists, UK

David Horne, Inverness Medical, UK

David Mant, General Practice and Department of Primary Health Care, University of Oxford, UK

Doris-Ann Williams, British In Vitro Diagnostics Association, UK

George Zajicek, Axis-Shield Diagnostics Ltd, UK

Hanns Christian Müller, Roche Diagnostics Ltd, Switzerland

Iñaki Gutiérrez Ibarluzea, Basque Office for Health Technology Assessment, Spain

Jag Grewal, Beckman Coulter United, UK

Janet Hiller, Adelaide Health Technology Assessment, Australia

Jeremy Moss, Roche Diagnostics Ltd, UK

Johan Wallin, Swedish Council on Technology Assessment in Health Care, Sweden

John Clarkson, Atlas Genetics Ltd, UK

Matthew Helbert, Department of Immunology, Directorate of Laboratory Medicine, Manchester, UK

Paul Glasziou, Centre for Evidence Based Medicine, University of Oxford, UK

Philip Wood, Consultant to the Diagnostics Industry

Richard Mayon-White, Department of Primary Health Care and Public Health, University of Oxford, UK

Susannah Fleming, Department of Engineering Science, University of Oxford, UK

Tammy Clifford, Canadian Agency for Drugs and Technologies in Health, Canada

Thierry Buclin, Department of Medicine, University Hospital of Lausanne, Switzerland

## Pre-publication history

The pre-publication history for this paper can be accessed here:

http://www.biomedcentral.com/1472-6963/10/109/prepub
